# Intensive Care Unit Admissions Purchased or Delivered by Veterans in the VA Health Care System

**DOI:** 10.1001/jamahealthforum.2025.5605

**Published:** 2025-12-12

**Authors:** Zachary Hahn, Hiam Naiditch, Victor Talisa, Brian Tyler, John R. Hotchkiss, Bryan J. McVerry, Sachin Yende, Florian B. Mayr

**Affiliations:** 1 VA Maine Togus Medical Center, Augusta; 2 VA Pittsburgh Healthcare System, Pittsburgh, Pennsylvania; 3 Division of Pulmonary, Allergy, Critical Care, and Sleep Medicine, Department of Medicine, University of Pittsburgh School of Medicine, Pittsburgh, Pennsylvania; 4 Clinical Research, Investigation, and Systems Modeling of Acute Illness (CRISMA) Center, University of Pittsburgh School of Medicine, Pittsburgh, Pennsylvania; 5 Office of Integrated Veteran Care, Veterans Health Administration, Washington, DC

## Abstract

**Importance:**

The Maintaining Internal Systems and Strengthening Integrated Outside Networks (MISSION) Act expanded access to community-based health care for veterans enrolled in the Veterans Affairs (VA) Health Care System. At the same time, the COVID-19 pandemic created unprecedented demand for intensive care unit (ICU) care. The combined impact of these changes on critical care delivery, outcomes, and spending remains unclear.

**Objective:**

To describe patterns in ICU admissions, case complexity, 90-day mortality, and VA spending for VA-delivered vs VA-purchased community ICU care from 2019 to 2023.

**Design, Setting, and Participants:**

Repeated cross-sectional study of 1 151 915 ICU admissions among veterans treated at 99 VA medical centers (VAMCs) and 4288 community hospitals reimbursed through the VA Community Care Network from January 1, 2019, to December 31, 2023. Stratified time series analyses were used to illustrate trends across 4 periods: pre-MISSION, post-MISSION, COVID-19 emergency, and post–COVID-19 stabilization. Interrupted time series analyses were then applied using unified regression models with interaction terms to assess differences across these periods.

**Main Outcomes and Measures:**

Main outcomes were monthly ICU admission volume, Case Mix Index weight, Charlson Comorbidity Index, 90-day all-cause mortality, and VA expenditures on community ICU care. Models were adjusted for demographic, clinical, and temporal covariates.

**Results:**

Of 1 151 915 ICU admissions among VA-enrolled veterans, including 270 237 at 99 VAMCs and 881 678 at 4288 community hospitals reimbursed through the VA community care network, 881 678 occurred in community hospitals. From 2019 to 2023, ICU admissions decreased by 21.3% in VAMCs and increased by 46.8% in community hospitals. Interrupted time series models showed increased mortality during the COVID-19 period in both settings and decreased mortality in VAMCs following the pandemic. Stratified time series models showed a postpandemic adjusted mortality rate of 18.4% (95% CI, 18.2%-18.7%) at VAMCs compared with 20.0% (95% CI, 19.8%-20.2%) in the community. Community hospitals had higher median Case Mix Indices, and total inflation-adjusted VA spending on community ICU care increased by 50% from $2.70 billion in 2019 to $4.04 billion in 2023, driven by increased admission volume.

**Conclusions and Relevance:**

This study found that VA-purchased community ICU care expanded markedly while incurring higher costs and slightly worse outcomes. These shifts underscore growing reliance on external critical care infrastructure and raise policy concerns around VA capacity planning, care integration, and quality oversight for veterans with high-acuity needs.

## Key Points

**Question:**

How did veteran intensive care unit (ICU) admission volume, patient complexity, spending, and 90-day mortality differ between Veterans Affairs (VA) medical centers and community hospitals following the VA MISSION (Maintaining Internal Systems and Strengthening Integrated Outside Networks) Act and during the COVID-19 pandemic?

**Findings:**

In this cross-sectional study of a national cohort of 1 151 915 veteran ICU admissions from 2019 to 2023, VA medical center ICU admissions decreased by 21.3%, while community ICU use increased by 46.8%. The shift toward community ICU care was associated with higher acuity, increased 90-day mortality, and a 50% increase in spending.

**Meaning:**

Results of this study suggest that careful reassessment of how VA and community ICU resources are balanced to ensure high-quality, sustainable critical care for veterans is warranted.

## Teaser

This cross-sectional study examines impacts of implementation of the Veterans Affairs MISSION (Maintaining Internal Systems and Strengthening Integrated Outside Networks) Act and the COVID-19 pandemic in critical care delivery for veterans comparing intensive care unit admissions at Veterans Affairs medical centers with community hospitals.

## Introduction

Intensive care services are a substantial and growing part of US health care spending, driven by an aging population, increased chronic illness, and advances in life-supporting technology.^1,2,3^ The veteran population is particularly affected because of higher rates of service-related illness and multimorbidity^4,5^ and relies heavily on the Veterans Affairs (VA) Health Care System, one of the largest and most geographically distributed integrated health care systems in the country. The VA Health Care System has greatly expanded the purchase of care from community hospitals since 2019, but little is known about the utilization of community intensive care services.

The VA Maintaining Internal Systems and Strengthening Integrated Outside Networks (MISSION) Act, implemented in 2019, consolidated multiple VA community-care programs into a single national network, aiming to reduce wait times and travel burdens for veterans.^6^ Subsequent evaluations have largely focused on outpatient access and system-level hospital metrics—clinic backlogs, specialty referrals, readmissions, and emergency department returns^7,8,9,10,11,12^—and condition-specific reports for select medical or surgical health cohorts.^13,14^ The COVID-19 pandemic then imposed a simultaneous supply and demand shock, straining critical care capacity and redirecting referrals across VA and non-VA hospitals.^15,16^ To our knowledge, it has not been assessed nationally whether these intersecting forces have altered the volume, acuity, outcomes, and cost of intensive care services delivered in VA medical centers (VAMCs) compared with those purchased from community hospitals.

We conducted a repeated cross-sectional study to examine the impact of the implementation of the VA MISSION Act and the COVID-19 pandemic on critical care delivery. The intensive care unit (ICU) admissions at VAMCs were compared with those purchased from community hospitals within 4 predefined periods: pre-MISSION, post-MISSION, COVID-19 emergency, and post–COVID-19 stabilization. For each period, we quantified changes in admission volume, case mix, 90-day mortality, and VA spending, hypothesizing that community hospitals would absorb an increasing share of ICU volume with corresponding differences in acuity and outcomes. Our findings aim to inform VA and national decision-makers by clarifying the critical trade-offs between the policy goal of expanded community access and the observed realities of higher costs and adverse clinical outcomes in those settings.

## Methods

### Study Design and Setting

We conducted a repeated cross-sectional study of ICU admissions among US veterans between January 1, 2019, and December 31, 2023. Each month served as a distinct cross-section, capturing all ICU admissions during that interval and enabling analyses of trends over time. This period was selected to include the years following the implementation of the VA MISSION Act and to span the COVID-19 pandemic. This start date avoids known data limitations in earlier community care network (CCN) claims, isolating the analysis from the confounding effects of prior policy eras.^17^ The study was deemed exempt from review by the VA Pittsburgh Institutional Review Board (IRB) and the Veterans IRB Network of Northern New England. We report our study in accordance with the Strengthening the Reporting of Observational Studies in Epidemiology (STROBE) reporting guideline.^18^

### Data Sources and Cohort Construction

We constructed the study cohort using data from 2 national repositories: the VA Corporate Data Warehouse for care at VAMCs and the Integrated Veteran Care dataset for VA-purchased community care.^8,19^ We identified ICU encounters using specialty bed-section codes within VAMCs and ICU-specific revenue center codes on institutional claims in the community.^20,21^ We excluded admissions if they lacked a Diagnosis-Related Group (DRG) code, reported zero ICU days, or had erroneous dates, such as negative time to death.

### Outcome Measures and Covariates

The primary outcomes were monthly ICU admission volume, case complexity, 90-day all-cause mortality, and annual VA spending on community ICU care. We categorized ICU admissions as either medical or surgical based on DRG code groupings^22^; for DRG-stratified analyses, encounters labeled as other were excluded. Case complexity was measured using 2 proxies: the DRG weight, reflecting acute severity,^23^ and the Charlson Comorbidity Index (CCI)^24^ as a measure of chronic disease burden. The CCI was calculated using the Quan *International Classification of Diseases*, *Tenth Revision*, algorithm^25^ applied to all VA and VA-purchased care in the 5 years before ICU admission. Mortality was ascertained from the VA Vital Status File and defined as death within 90 days of the hospital admission date. Spending was defined as the total amount paid by VA for institutional and professional claims associated with each community ICU admission, from hospital admission through discharge.^26^ While VA reimbursement for community care is often capped at local Medicare rates, this cap is not always binding; payments may exceed Medicare rates in cases of negotiated agreements or for certain emergency care episodes. Therefore, the spending amounts in this study reflect actual paid claims, not predetermined rate ceilings. Spending amounts are presented in nominal dollars and adjusted to 2023 US dollars using the Bureau of Economic Analysis gross domestic product deflator.^27,28^ Spending analyses were limited to community ICU hospitalizations, as patient-level cost data are unavailable for VA-delivered ICU care. Race and ethnicity data were obtained from the VA Corporate Data Warehouse, where race and ethnicity are self-reported by veterans at the time of enrollment. Options are defined by the participant and recorded by VA administrative staff. Race and ethnicity were included to characterize the study population.

### Statistical Analysis

We used descriptive statistics to summarize baseline patient characteristics and unadjusted trends in ICU admissions, patient complexity, costs, and outcomes. Continuous variables are presented as medians with IQRs or means with SDs, and categorical variables are presented as frequencies and percentages. To explore subgroup differences, we stratified key analyses by DRG group (medical vs surgical).

We conducted a stratified time series analysis, estimating separate linear regression models for each period and facility type (VA vs community) to assess temporal trends within each major policy and public health period. Four prespecified periods were defined a priori: pre-MISSION (January-May 2019), post-MISSION (June 2019-February 2020), COVID-19 emergency (March 2020-April 2022), and post–COVID-19 stabilization (May 2022-December 2023). Outcomes were aggregated by month. The eMethods in Supplement 1 presents additional details.

Primary regression models estimated trends independently within each period using separate slope and intercept terms to characterize within-period dynamics and assess divergences between VA and CCN ICUs. However, this stratified regression does not permit formal statistical comparisons across periods. Therefore, as a sensitivity analysis, we conducted an interrupted time series analysis using unified models incorporating interaction terms for policy breakpoints to describe differences associated with the implementation of the MISSION Act and the COVID-19 pandemic.^10^ Interrupted time series estimates changes in level and trend across periods, enabling the evaluation of dynamic system responses to discrete interventions or disruptions.^10,29^

For adjusted 90-day mortality trends, we used a 2-stage approach.^30^ First, we fit multivariable logistic regression models at the patient level to estimate predicted 90-day mortality, adjusting for demographic characteristics, rural residence, hospital and ICU length of stay, DRG weight, CCI, and facility type. We then averaged the predicted probabilities, which were aggregated by month and facility type, to generate monthly risk-adjusted estimates of 90-day mortality. These estimates were then modeled using stratified linear regression within the 4 periods.

Model-based trend lines illustrate estimated patterns within each period but should not be interpreted as statistically significant differences between periods without formal statistical testing. Analyses were conducted using R version 4.4.1 (R Foundation for Statistical Computing).

### Supplementary System-Level Analysis

To contextualize VA-purchased ICU care within national hospital utilization patterns, we conducted supplementary analyses of ICU bed availability, occupancy rates, and the VA’s share of non-Medicare/Medicaid ICU bed-days using RAND hospital data from 2019 to 2023^31^ and Centers for Disease Control and Prevention/Department of Health and Human Services data during the mandated reporting period from 2020 to 2023.^32^ The full methods for these analyses are provided in the eMethods in Supplement 1.

## Results

### Study Population and Cohort Characteristics

Between January 1, 2019, and December 31, 2023, there were 1 151 915 ICU admissions among VA-enrolled veterans, including 270 237 at 99 VAMCs and 881 678 at 4288 community hospitals reimbursed through the VA CCN (eFigure 1 in Supplement 1). Medical admissions accounted for 69.5% of ICU volume across both settings. The patients in the CCN ICUs were older (median age, 72 [IQR, 64–77] years vs 71 [IQR, 63–76] years) and more likely to reside in rural areas (39.3% vs 27.7%) compared with those in VAMCs. The patients in the CCN were more often White (76.9% vs 68.6%), while VAMCs served more Black veterans (24.5% vs 14.9%). In comparison, the rates for Asian patients were 0.52% for CCN and 0.54% for VAMC and for Hispanic patients were 4.7% for CCN and 6.6% for VAMC. The mean (SD) CCI was higher among VAMC patients (5.0 [3.6] vs 4.2 [3.3]). The Case Mix Index (CMI), which reflects illness severity, was higher for CCN admissions (median, 1.83 [IQR, 1.16–2.55] vs 1.63 [IQR, 1.06–2.14]), particularly for surgical cases. The VAMC patients had longer hospital stays (median, 6 [IQR, 4–11] days vs 5 [IQR, 2–9] days) but shorter ICU stays (median 2 [IQR, 2–4] days vs 3 [IQR, 2–6] days) (Table; eTable 1 in Supplement 1). Missingness of covariates was low across all variables (eTable 11 in Supplement 1).

**Table.** Baseline Characteristics of Veteran ICU Admissions by Setting, 2019-2023

Characteristic	Setting, No. (%)		
	All	VAMC	CCN hospitals
Admissions	1 151 915 (100)	270 237 (23.5)	881 678 (76.5)
Medical admissions	800 778 (69.5)	193 623 (71.7)	607 155 (68.9)
Unique patients	726 666 (100)	205 596 (28.3)	579 336 (79.7)
Age, median (IQR), y	71 (63-77)	71 (63-76)	72 (64-77)
Sex			
Female	54 832 (4.8)	13 546 (5.0)	41 286 (4.7)
Male	1 097 083 (95.2)	256 691 (95.0)	840 392 (95.3)
Race and ethnicity^a^			
African American/Black	194 457 (17.2)	65 394 (24.5)	129 063 (14.9)
Asian	5944 (0.53)	1434 (0.54)	4510 (0.52)
Hispanic	58 331 (5.1)	17 696 (6.6)	40 635 (4.7)
White	848 436 (74.9)	183 266 (68.6)	665 170 (76.9)
Married	553 628 (48.1)	122 156 (45.2)	431 472 (48.9)
Rural residence	421 083 (36.6)	74 854 (27.7)	346 229 (39.3)
CCI, mean (SD)	4.38 (3.41)	5.01 (3.57)	4.18 (3.33)
CMI, median (IQR)	1.78 (1.14-2.53)	1.63 (1.06-2.14)	1.83 (1.16-2.55)
Medical CMI, median (IQR)	1.34 (0.98-1.86)	1.27 (0.91-1.85)	1.35 (0.99-1.87)
Surgical CMI, median (IQR)	3.49 (2.34-5.06)	3.03 (2.03-4.50)	3.73 (2.41-5.11)
ICU LOS, median (IQR), d	3 (2-6)	2 (2-4)	3 (2-6)
Hospital LOS, median (IQR), d	5 (3-9)	6 (4-11)	5 (2-9)
Area Deprivation Index, median (IQR)	62 (41-81)	60 (38-80)	63 (42-81)
In-hospital mortality	83 672 (7.3)	21 948 (8.1)	61 724 (7.0)
90-d Mortality	228 862 (19.9)	52 224 (19.3)	176 638 (20.0)

Abbreviations: CCI, Charlson Comorbidity Index; CCN, community care network; CMI, Case Mix Index (Diagnosis-Related Group weight); ICU, intensive care unit; LOS, length of stay; VAMC, Veterans Affairs Medical Center.

^a^ Race and ethnicity data were obtained from the VA Corporate Data Warehouse, where race and ethnicity are self-reported by veterans at the time of enrollment. Options are defined by the participant and recorded by VA administrative staff. Race and ethnicity were included to characterize the study population.

Care pathways and discharge patterns differed. The patients in the CCN ICUs were more often transferred from other facilities or admitted via emergency departments, while VAMCs treated more direct or elective cases. The patients in the CCN had higher discharge rates to skilled nursing facilities or hospice (eTables 2 and 3 in Supplement 1).

### ICU Admission Volume

**Figure 1.**
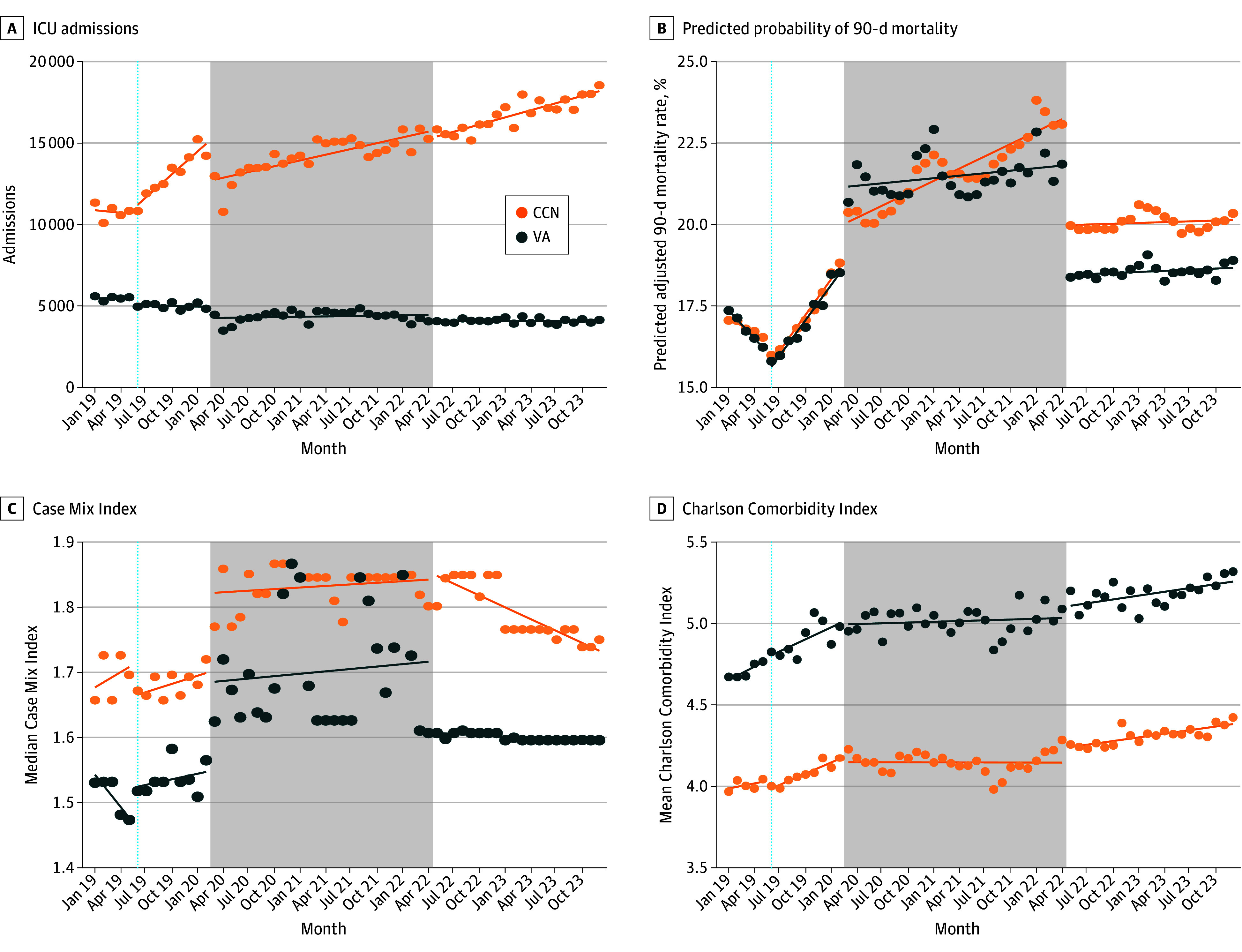
Stratified Time Series Trends in Veteran Intensive Care Utilization and Outcomes, 2019-2023 Dashed vertical line indicates VA MISSION Act implementation (June 2019); gray band, COVID-19 emergency (March 2020-April 2022). Trend lines are model based; dots represent monthly observations. CCN indicates community care network; ICU, intensive care unit; MISSION, Maintaining Internal Systems and Strengthening Integrated Outside Networks; VA, Veterans Affairs.

ICU admissions diverged between settings over time (Figure 1A). CCN hospitals experienced a sharp increase following the implementation of the MISSION Act, followed by a transient decrease during the early stages of the pandemic and then sustained near linear growth. In contrast, VAMC ICU admissions decreased steadily across all periods without evidence of a COVID-19–related surge. Overall, VAMC ICU admissions decreased from 62 982 in 2019 to 49 584 in 2023, while CCN admissions increased from 143 030 to 209 953. By 2023, CCN hospitals accounted for more than 80% of VA-financed ICU admissions.

### Mortality

Adjusted 90-day mortality fluctuated substantially during the COVID-19 period in both systems (Figure 1B). From the end of the MISSION period to the onset of the COVID-19 period, mortality increased by 5.87 percentage points (95% CI, 5.24-6.50 percentage points) among patients in the VAMC ICUs and 4.55 percentage points (95% CI, 3.92-5.18 percentage points) in community hospital ICUs based on unified model estimates (eTable 7 in Supplement 1). A postpandemic level shift of −2.70 percentage points (95% CI, −3.16 to −2.23 percentage points) was observed among VAMCs, while mortality in CCN hospitals remained stable. During the post–COVID-19 period, stratified time series estimates indicated a mortality rate of 18.4% (95% CI, 18.2%-18.7%) at VAMCs and 20.0% (95% CI, 19.8%-20.2%) in CCN facilities. These levels reflect the modeled post–COVID-19 mortality intercepts derived from the segmented regression results depicted in Figure 1.

### Case Complexity

Stratified time series models demonstrated that the median CMI remained consistently higher in community hospitals within each policy period, with postpandemic differences of 1.61 (95% CI, 1.59-1.62) at VAMCs vs 1.85 (95% CI, 1.84-1.87) in community hospitals. Similarly, the mean CCI was higher among patients in the VAMC ICUs within each period and increased during the pandemic across both settings, with postpandemic differences of 5.10 (95% CI, 5.04-5.15) at VAMCs vs 4.23 (95% CI, 4.18-4.28) in the community (Figure 1C and D).

Unified model estimates confirmed significant upward shifts in CMI and CCI associated with the onset of the COVID-19 period, reflecting an increase in patient complexity over time (eTables 8 and 9 in Supplement 1). Postpandemic, both measures stabilized but remained elevated, particularly in community hospitals. Model estimates for ICU admissions, mortality, case complexity, and comorbidity trends, including DRG-stratified analyses, are presented in eFigures 2 and 3 and eTables 6 to 9 in Supplement 1.

### VA Spending on Community ICU Care

**Figure 2.**
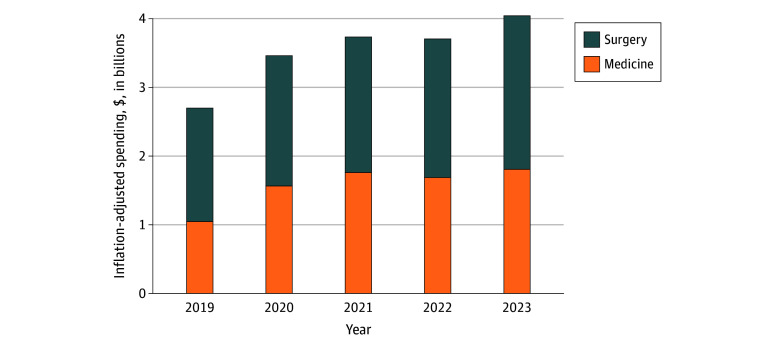
Inflation-Adjusted VA Spending on Community ICU Care by Admission Type, 2019-2023 Annual VA expenditures for CCN ICU admissions, adjusted to 2023 US dollars using the GDP price deflator. Costs are stratified by DRG specialty (medical or surgical) and shown in billions of dollars. Each bar represents total adjusted spending per year. CCN indicates community care network; DRG, Diagnosis-Related Group; GDP, gross domestic product; ICU, intensive care unit; VA, Veterans Affairs.

Financial trends mirrored these shifts (Figure 2; eTable 10 in Supplement 1). Inflation-adjusted VA spending for community ICU care increased by approximately 50%, from $2.70 billion to $4.04 billion between 2019 and 2023. Mean (SD) inflation-adjusted per-admission costs for surgical ICU stays decreased from $48 319 ($68 017) in 2019 to $44 881 ($52 716) in 2023. In contrast, mean (SD) inflation-adjusted costs for medical ICU admissions remained constant after peaking at $18 509 ($20 503) in 2021 during the COVID-19 period (eTables 4 and 5 in Supplement 1).

### VA Community ICU Expansion and System-Level Utilization Trends

**Figure 3.**
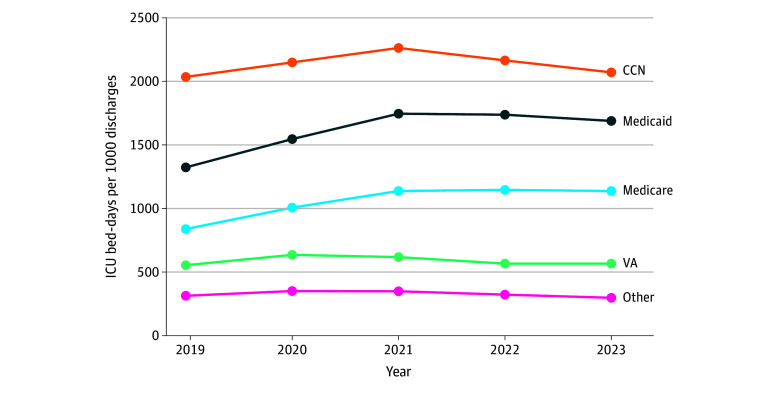
ICU Bed-Days Per 1000 Discharges by Payer Type and System, 2019-2023 ICU bed-days per 1000 discharges reflect the intensity of ICU utilization across systems. CCN values represent VA-purchased ICU care in community hospitals. Medicare and Medicaid estimates were derived from RAND Hospital Data. Other includes ICU bed-days not covered by Medicare, Medicaid, or the VA. CCN indicates community care network; ICU, intensive care unit; VA, Veterans Affairs.

From 2019 to 2023, VA-purchased ICU care increased steadily, while the total number of ICU admissions delivered within VAMCs decreased. However, ICU utilization intensity within VAMCs, measured as ICU bed-days per 1000 hospital discharges, remained relatively stable, ranging from 500 to 600. In contrast, VA-purchased ICU utilization at CCN hospitals consistently exceeded 2000 ICU bed-days per 1000 discharges, reflecting markedly higher intensity of use, with modest increases during the pandemic (Figure 3).

**Figure 4.**
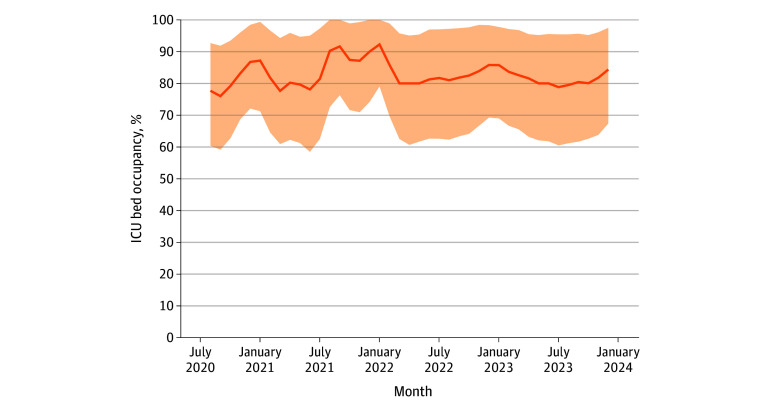
Monthly ICU Bed Occupancy Among Community Hospitals Providing VA-Purchased Care This figure illustrates the median monthly ICU occupancy rates, based on hospital reports of staffed ICU beds submitted to the HHS Protect Public Data Hub. Staffed beds reflect those that are set up, staffed, and immediately available for patient care. Data are limited to community hospitals that submitted VA CCN claims. The shaded area indicates IQRs of facility-level occupancy. ICU occupancy above 85% is commonly cited as the threshold associated with strained capacity. CCN indicates community care network; HHS, Department of Health and Human Services; ICU, intensive care unit; VA, Veterans Affairs.

The median occupancy of licensed ICU beds in community hospitals increased from 55.8% (IQR, 40.0%-71.3%) in 2019 to 64.0% (IQR, 47.8%-78.7%) in 2021, before declining to 56.2% (IQR, 38.8%-72.9%) by 2023. Over the same period, median VAMC ICU occupancy, based on licensed beds, ranged from 45.0% (95% CI, 44.4%-46.5%) to 51.5% (95% CI, 51.1%-52.1%), with little change in ICU admission thresholds (eTable 12 in Supplement 1). Median staffed occupancy peaked at 83.6% (IQR, 69.5%-96.9%) in 2021 and remained elevated at 81.1% (IQR, 65.0%-94.9%) in 2023. Between 2019 and 2023, VA-purchased ICU bed-days increased from 662 091 to 1 014 921, increasing VA’s share of national non-Medicare/Medicaid ICU bed-days from 11.6% to 17.2%. In contrast, VAMC-delivered ICU bed-days decreased modestly (Figure 4; eTable 13 in Supplement 1).

## Discussion

In this national cohort study, the delivery of critical care within the VA health care system shifted from VAMCs to community hospitals, following the implementation of the MISSION Act and the COVID-19 pandemic. By 2023, more than 80% of VA-financed ICU admissions occurred in community hospitals. This transition coincided with increased spending, increasing illness severity, and persistently higher adjusted 90-day mortality among veterans receiving care in CCN hospitals compared with VAMCs. These trends have important implications for VA policy, fiscal sustainability, and health system capacity.

The MISSION Act expanded community care eligibility to improve access for VA-enrolled veterans facing geographic or logistical barriers to VA services. Community ICU use increased steadily following implementation and accelerated during the pandemic. This growth coincided with decreasing VAMC ICU admissions, suggesting that community care is increasingly substituted for in-house capacity. This net shift raises concerns that VAMC ICU infrastructure may be underutilized, potentially weakening institutional capability over time. Reinvestment in VA ICU infrastructure may be crucial to preserving access, improving outcomes, and sustaining operational readiness. The trade-off, expanding access through community care while in-house ICU volume contracts, parallels the Military Health System’s earlier pivot toward outsourcing, which ultimately eroded clinical readiness.^33,34,35,36,37^ The VA faces similar risks unless current trends are addressed, underscoring the need to balance short-term access gains with long-term institutional resilience. While our findings underscore the importance of preserving VA ICU capacity, the optimal mechanisms to achieve this, whether through infrastructure reinvestment, operational realignment, or alternative models, remain uncertain and merit further study.

VA spending on community ICU care increased by approximately 50%, from $2.70 billion to $4.04 billion over the study period. Although inflation-adjusted per-admission costs were stable or decreasing, increasing volume drives concerns about long-term sustainability and opportunity costs for other priorities, including specialty care, mental health services, and infrastructure.

Adjusted 90-day mortality was consistently lower for veterans treated in VAMCs, particularly among surgical patients. While causality cannot be inferred from observational data, these findings align with prior studies showing equal or superior outcomes with VA-delivered care.^38,39,40^ Potential drivers include the VA’s integrated delivery model, embedded care teams, and postdischarge continuity, features that are difficult to replicate in fragmented community networks, particularly for patients with high-acuity needs.

These trends underscore growing concerns for VA preparedness and national ICU resilience. Community hospitals delivering VA-purchased ICU care exhibited median occupancy rates exceeding 80% in recent years, based on staffed bed counts. In contrast, VAMC occupancy based on licensed beds remained below 52%. Because licensed bed counts often overstate true operational capacity, actual VAMC occupancy likely falls between these extremes.^1^ This discrepancy suggests latent capacity within the VA that could be strategically leveraged to alleviate national ICU strain. Optimal ICU occupancy is generally considered to range between 70% and 75% to maintain surge flexibility and quality of care.^41^ Sustained occupancy above this level, particularly more than 85%, has been associated with delays in care, emergency department boarding, and increased mortality.^42,43,44,45^ Our findings build on prior data, which show that staffed hospital bed occupancy averaged 75.3% after the pandemic, driven by a 16% reduction in available beds.^46^ States with the largest veteran populations, including California, Texas, Florida, North Carolina, and Pennsylvania, exceeded this national average.^47^ In this context, underutilized VAMC capacity presents both a challenge and a strategic opportunity to enhance national critical care resilience.

This imbalance is reflected in the intensity of ICU utilization. CCN hospitals consistently delivered more than 2000 ICU bed-days per 1000 discharges, nearly 4 times the rate observed in VAMCs. As VA-purchased ICU admissions continue to shift into community settings, the risk of exacerbating system strain and displacing care for nonveterans grows. Rebalancing this load by optimizing in-house ICU capacity is crucial to maintaining access for veterans and enhancing resilience across both VA and civilian systems.

Within VA, decreasing ICU volumes may undermine core institutional functions. Lower caseloads reduce opportunities for clinical training, procedural proficiency, and emergency response capacity under the Fourth Mission.^48^ The VA trains more than 120 000 health professionals annually, many of whom rotate through VAMCs for critical care education.^49^ Sustained high-acuity exposure is essential for developing and maintaining workforce competency, operational readiness, and national emergency capability. Diminished volumes may also limit institutional research, quality improvement, and evidence-generation activities, while contributing to staff attrition due to reduced professional growth opportunities.^50^ Importantly, a broad body of evidence links higher patient volumes with improved outcomes for conditions such as sepsis, mechanical ventilation, and postoperative care.^51^ As policymakers consider the future of VA critical care delivery, it is essential to recognize that decreasing inpatient volumes risk eroding clinical performance and institutional resilience.

Our findings also underscore the VA’s growing role in shaping national ICU capacity. By 2023, VA-purchased CCN ICU bed-days accounted for 17.2% of all non-Medicare/Medicaid ICU bed-days nationally, up from 11.6% in 2019. This expansion reflects VA’s growing footprint not only as a payer but also as a major contributor to ICU workloads in community hospitals. Coordinated capacity planning across systems and alignment between VA policies and broader health infrastructure will be essential to maintain ICU resilience.

Distinguishing between payer shift and true access gains remains a critical challenge. Some increase in CCN ICU use may reflect reclassification of care previously financed by Medicare, now paid by the VA under expanded eligibility.^52^ Without full payer data, this distinction remains uncertain. If the MISSION Act primarily shifted payment sources, cost increases are real but access gains may be overstated. Conversely, if policy expanded timely ICU access for underserved veterans, higher costs may be justified, provided quality is maintained.

We do not interpret our findings as a blanket indictment of community care. For many veterans, CCN ICU access offers meaningful benefits, including proximity, reduced wait times, or alignment with personal preferences. Community partnerships remain essential in areas where VA facilities are sparse or nearing capacity. However, current trends suggest that community care is no longer supplemental; it has become the dominant pathway for critical illness. Ensuring high-quality, cost-effective care will require robust quality monitoring, stronger contracting oversight, and accountability mechanisms across all settings.

### Limitations

Our study has limitations. First, our analyses are observational and cannot establish causality. Second, we lacked access to non-VA payer data, limiting visibility into care delivered outside VA authorization. Third, illness severity and comorbidity were measured using administrative codes and may not fully capture physiological acuity or social complexity. Fourth, differences in coding practices, particularly in community hospitals, could contribute to observed variation in case mix. Fifth, cost data were available only for community hospitalizations, precluding direct financial comparisons with VAMC care. Finally, despite careful model specification and stratification, residual confounding may persist, especially for outcomes affected by postdischarge disposition.

## Conclusions

In this study, delivery of critical care to VA-enrolled veterans has shifted toward community hospitals, driven by policy changes and pandemic disruptions. This transition has coincided with increasing costs, growing dependence on strained community systems, and outcome differences favoring VA-delivered ICU care. To maintain access and system resilience, the VA must preserve sufficient in-house ICU capacity and hold community partners to high standards. Strengthening VA’s role as a full-service, integrated health system will require investments in infrastructure, oversight, and cross-system coordination to meet the evolving needs of veterans and the broader health system.

## Notes

Supplement 1. **eMethods**. **eTable 1.** Baseline Characteristics of Veteran ICU Admissions by Setting and Year **eTable 2.** Admission Source by Facility Type **eTable 3.** Disposition at Hospital Discharge by Facility Type **eTable 4.** Medical ICU Admissions, Nominal Costs, Length of Stay, Case Mix, and Mortality by Facility Type **eTable 5.** Surgical ICU Admissions, Nominal Costs, Length of Stay, Case Mix, and Mortality by Facility Type **eTable 6.** Segmented Interrupted Time Series Regression Estimates for Monthly ICU Admissions, by Facility Type and DRG Group **eTable 7.** Segmented Interrupted Time Series Regression Estimates for 90-day Mortality, by Facility Type and DRG Group **eTable 8.** Segmented Interrupted Time Series Regression Estimates for Monthly Case Mix Index, by Facility Type and DRG Group **eTable 9.** Segmented Interrupted Time Series Regression Estimates for Monthly Charlson Comorbidity Index, by Facility Type and DRG Group **eTable 10.** Nominal and Inflation-adjusted costs using the Bureau of Economic Gross Domestic Product deflator **eTable 11.** Absolute and Relative Missingness for Study Covariates by Year and Facility Type **eTable 12.** VAMC ICU Admission Rates by DRG Group and Year **eTable 13.** VA-Purchased ICU Bed-Days as a Proportion of National Non-Medicare/Medicaid ICU Use and Trends in ICU Occupancy, 2019–2023 **eFigure 1.** Cohort derivation for VA-purchased or delivered ICU admissions between 2016 and 2023. **eFigure 2.** Interrupted time-series trends in veteran medical intensive care utilization and outcomes, 2019-2023 **eFigure3.** Interrupted time-series trends in veteran surgical intensive care utilization and outcomes, 2019-2023

Supplement 2. Data Sharing Statement
