# Supplementary material for: Intensive Care Unit Admissions Purchased or Delivered by Veterans in the VA Health Care System
Source: JAMA Health Forum. 2025 Dec 12;6(12):e255605. doi: 10.1001/jamahealthforum.2025.5605 (PMC12701510; doi:10.1001/jamahealthforum.2025.5605)
Supplement: Supplement 1. — eMethods. eTable 1. Baseline Characteristics of Veteran ICU Admissions by Setting and Year eTable 2. Admission Source by Facility Type eTable 3. Disposition at Hospital Discharge by Facility Type eTable 4. Medical ICU Admissions, Nominal Costs, Length of Stay, Case Mix, and Mortality by Facility Type eTable 5. Surgical ICU Admissions, Nominal Costs, Length of Stay, Case Mix, and Mortality by Facility Type eTable 6. Segmented Interrupted Time Series Regression Estimates for Monthly ICU Admissions, by Facility Type and DRG Group eTable 7. Segmented Interrupted Time Series Regression Estimates for 90-day Mortality, by Facility Type and DRG Group eTable 8. Segmented Interrupted Time Series Regression Estimates for Monthly Case Mix Index, by Facility Type and DRG Group eTable 9. Segmented Interrupted Time Series Regression Estimates for Monthly Charlson Comorbidity Index, by Facility Type and DRG Group eTable 10. Nominal and Inflation-adjusted costs using the Bureau of Economic Gross Domestic Product deflator eTable 11. Absolute and Relative Missingness for Study Covariates by Year and Facility Type eTable 12. VAMC ICU Admission Rates by DRG Group and Year eTable 13. VA-Purchased ICU Bed-Days as a Proportion of National Non-Medicare/Medicaid ICU Use and Trends in ICU Occupancy, 2019–2023 eFigure 1. Cohort derivation for VA-purchased or delivered ICU admissions between 2016 and 2023. eFigure 2. Interrupted time-series trends in veteran medical intensive care utilization and outcomes, 2019-2023 eFigure3. Interrupted time-series trends in veteran surgical intensive care utilization and outcomes, 2019-2023 [file jamahealthforum-e255605-s001.pdf]

Supplemental Online Content

Hahn Z, Naiditch H, Talisa V, et al. Intensive care unit admissions purchased or delivered by veterans in the VA Health Care System. *JAMA Health Forum*. 2025;6(12):e255605. doi:10.1001/jamahealthforum.2025.5605

eMethods.
eTable 1. Baseline Characteristics of Veteran ICU Admissions by Setting and Year

eTable 2. Admission Source by Facility Type

eTable 3. Disposition at Hospital Discharge by Facility Type

eTable 4. Medical ICU Admissions, Nominal Costs, Length of Stay, Case Mix, and Mortality by Facility Type

eTable 5. Surgical ICU Admissions, Nominal Costs, Length of Stay, Case Mix, and Mortality by Facility Type

eTable 6. Segmented Interrupted Time Series Regression Estimates for Monthly ICU Admissions, by Facility Type and DRG Group

eTable 7. Segmented Interrupted Time Series Regression Estimates for 90-day Mortality, by Facility Type and DRG Group

eTable 8. Segmented Interrupted Time Series Regression Estimates for Monthly Case Mix Index, by Facility Type and DRG Group

eTable 9. Segmented Interrupted Time Series Regression Estimates for Monthly Charlson Comorbidity Index, by Facility Type and DRG Group

eTable 10. Nominal and Inflation-adjusted costs using the Bureau of Economic Gross Domestic Product deflator

eTable 11. Absolute and Relative Missingness for Study Covariates by Year and Facility Type

eTable 12. VAMC ICU Admission Rates by DRG Group and Year

eTable 13. VA-Purchased ICU Bed-Days as a Proportion of National Non-Medicare/Medicaid ICU Use and Trends in ICU Occupancy, 2019–2023

eFigure 1. Cohort derivation for VA-purchased or delivered ICU admissions between 2016 and 2023.

eFigure 2. Interrupted time-series trends in veteran medical intensive care utilization and outcomes, 2019-2023

eFigure3. Interrupted time-series trends in veteran surgical intensive care utilization and outcomes, 2019-2023

This supplemental material has been provided by the authors to give readers additional information about their work.

© 2025 Hahn Z et al. *JAMA Health Forum*.

ONLINE-ONLY SUPPLEMENT

SUPPLEMENTARY METHODS

System-Level Analyses

To contextualize the scale and policy implications of VA-purchased ICU care, we conducted system-level analyses of ICU capacity and utilization across U.S. hospitals from 2019 through 2023. These analyses integrated data from four primary sources: (1) RAND Hospital Data, (2) the VA Corporate Data Warehouse (CDW), (3) the VA Integrated Veteran Care (IVC) dataset, and (4) the HHS Protect Public Data Hub. Each source provided complementary perspectives on ICU infrastructure, occupancy trends, and VA's growing footprint within the U.S. critical care system.

RAND Hospital Data standardizes facility-level characteristics from CMS's Healthcare Cost Report Information System (HCRIS), including reported licensed ICU bed counts, ICU bed-days available (licensed beds × 365), ICU bed-days used, and occupancy rates.[1] ICU utilization is further stratified by payer type (Medicare, Medicaid, and non-Medicare/Medicaid), allowing for the estimation of the proportion of ICU care financed outside traditional entitlement programs. This enabled us to benchmark VA-purchased ICU care against broader non-Medicare/Medicaid ICU use nationally.[2]

To estimate ICU capacity and occupancy within VA Medical Centers (VAMCs), we used CDW daily census data. Licensed ICU bed counts at each VAMC were multiplied by 365 to derive annual ICU bed-days available. Daily occupied ICU bed counts were summed to calculate the total number of ICU bed-days used. Occupancy was calculated as the ratio of occupied to available ICU bed-days and summarized using median values and interquartile ranges across all VAMCs.[3]

Recognizing that licensed bed counts often overestimate functional ICU capacity, we supplemented these data with HHS Protect reporting.[4,5] HHS Protect aggregates weekly hospital-level reports on *staffed* ICU beds, i.e., those that are set up, staffed, and ready for immediate use, with corresponding occupancy counts. These data provide a more accurate measure of operational capacity. Because VA facilities do not report to HHS Protect, we restricted analysis to community hospitals that submitted ICU claims under the VA Community Care Network (CCN). Monthly staffed-bed occupancy rates were calculated and summarized using median values and interquartile ranges across CCN hospitals.

© 2025 Hahn Z et al. *JAMA Health Forum*.

Data Linkage and Utilization Metrics

Hospitals were linked across RAND, HHS Protect, and IVC datasets using National Provider Identifiers (NPIs) and Medicare Certification Numbers (CCNs), in accordance with established protocols.[6] From RAND, we extracted the annual number of ICU bed-days used, the number of ICU bed-days available, and payer-type distributions. From IVC, we summed ICU bed-days reimbursed by VA through the CCN program. These served as the numerator for calculating VA's share of non-Medicare/Medicaid ICU utilization. Denominators were constructed from RAND estimates of non-Medicare/Medicaid ICU bed-days used in community hospitals. From HHS Protect, we extracted weekly staffed ICU bed counts and occupied bed counts from all hospitals we were able to match with our IVC data. Monthly occupancy rates were derived and used to contextualize ICU strain in community settings. Taken together, these datasets enabled us to compare capacity, occupancy, and utilization across VAMCs and community hospitals, and to estimate VA's expanding footprint in national ICU care.

Time-Series Outcomes and Model Specification

To evaluate changes in ICU admission thresholds and patient complexity over time, we performed segmented time-series analyses using discharge-level data from VA CDW and IVC.

ICU admission thresholds were defined as the proportion of acute-care hospitalizations resulting in ICU admission, calculated exclusively for VA Medical Centers due to RAND's lack of admission-level data. ICU utilization intensity, a normalized measure of ICU use, was defined as ICU bed-days per 1,000 hospital discharges.

Patient complexity was assessed using Diagnosis-Related Group (DRG) weights and Charlson Comorbidity Index (CCI) scores. DRG weight distributions were right-skewed; therefore, the median DRG weight was aggregated monthly. In contrast, mean CCI was used for time-trend modeling due to the poor resolution and volatility of median CCI at the monthly level.

Each outcome (ICU use per discharge, median DRG weight, and mean CCI) was modeled using segmented ordinary least squares linear regression. Models included separate intercepts and slopes for each of the four predefined time periods (Pre-MISSION, MISSION Pre-COVID, MISSION-COVID, Post-COVID), with facility-level fixed effects and time-by-facility interaction terms to account for within-site trend variation.

© 2025 Hahn Z et al. *JAMA Health Forum*.

Limitations

These analyses relied on aggregate facility-reported data and claims-based encounters. RAND and CDW data reflect licensed beds, not real-time availability. HHS Protect does not include VA hospitals, limiting staffed-bed comparisons. Community hospital coding practices may differ from those of VAMCs, potentially leading to misclassification. We lacked access to non-VA payer data, which prevented a full characterization of ICU utilization outside of VA-financed episodes.

References

1. RAND Hospital Data. Accessed June 20, 2025. https://www.hospitaldatasets.org/
2. Halpern NA, Pastores SM. Critical Care Medicine Beds, Use, Occupancy, and Costs in the United States: A Methodological Review*. *Crit Care Med*. 2015;43(11):2452-2459. doi:10.1097/CCM.0000000000001227
3. Chen LM, Render M, Sales A, Kennedy EH, Wiitala W, Hofer TP. Intensive Care Unit Admitting Patterns in the Veterans Affairs Health Care System. *Arch Intern Med*. 2012;172(16):1220. doi:10.1001/archinternmed.2012.2606
4. Weekly United States Hospitalization Metrics by Jurisdiction, During Mandatory Reporting Period from August 1, 2020 to April 30, 2024, and for Data Reported Voluntarily Beginning May 1, 2024, National Healthcare Safety Network (NHSN) - ARCHIVED | Data | Centers for Disease Control and Prevention. Accessed August 6, 2025. https://data.cdc.gov/Public-Health-Surveillance/Weekly-United-States-Hospitalization-Metrics-by-Ju/aemt-mg7g/about_data
5. Leuchter RK, Delarmente BA, Vangala S, Tsugawa Y, Sarkisian CA. Health Care Staffing Shortages and Potential National Hospital Bed Shortage. *JAMA Netw Open*. 2025;8(2):e2460645. doi:10.1001/jamanetworkopen.2024.60645
6. National Provider Identifier (NPI) to Medicare CCN Crosswalk. NBER. Accessed June 23, 2025. https://www.nber.org/research/data/national-provider-identifier-npi-medicare-ccn-crosswalk

© 2025 Hahn Z et al. *JAMA Health Forum*.

SUPPLEMENTARY TABLES AND FIGURES

1. eTable 1. Baseline Characteristics of Veteran ICU Admissions by Setting and Year

2. eTable 2. Admission Source by Facility Type

3. eTable 3. Disposition at Hospital Discharge by Facility Type

4. eTable 4. Medical ICU Admissions, Nominal Costs, Length of Stay, Case Mix, and Mortality by Facility Type

5. eTable 5. Surgical ICU Admissions, Nominal Costs, Length of Stay, Case Mix, and Mortality by Facility Type

6. eTable 6. Segmented Interrupted Time Series Regression Estimates for Monthly ICU Admissions, by Facility Type and DRG Group

7. eTable 7. Segmented Interrupted Time Series Regression Estimates for 90-day Mortality, by Facility Type and DRG Group

8. eTable 8. Segmented Interrupted Time Series Regression Estimates for Monthly Case Mix Index, by Facility Type and DRG Group

9. eTable 9. Segmented Interrupted Time Series Regression Estimates for Monthly Charlson Comorbidity Index, by Facility Type and DRG Group

10. eTable 10. Nominal and Inflation-adjusted costs using the Bureau of Economic Gross Domestic Product deflator

11. eTable 11. Absolute and Relative Missingness for Study Covariates by Year and Facility Type

12. eTable 12. VAMC ICU Admission Rates by DRG Group and Year

13. eTable 13. VA-Purchased ICU Bed-Days as a Proportion of National Non-Medicare/Medicaid ICU Use and Trends in ICU Occupancy, 2019–2023

1. eFigure 1. Cohort derivation for VA-purchased or delivered ICU admissions between 2016 and 2023

2. eFigure 2. Interrupted time-series trends in veteran medical intensive care utilization and outcomes, 2019-2023

3. eFigure3. Interrupted time-series trends in veteran surgical intensive care utilization and outcomes, 2019-2023

© 2025 Hahn Z et al. *JAMA Health Forum*.

eTable1. Baseline Characteristics of Veteran ICU Admissions by Facility Type and Year

Facility	Measure	2019	2020	2021	2022	2023
All	Admissions	206,012	215,458	232,074	238,834	259,537
VA	Admissions	62,982	53,268	54,781	49,622	49,584
VA	Medicine Admissions	43,513 (69.09)	38,520 (72.31)	39,596 (72.28)	36,152 (72.85)	35,842 (72.29)
VA	Patients	54,483	46,752	47,919	43,417	43,106
VA	Age	70 (63, 75)	70 (63, 75)	71 (63, 76)	72 (63, 76)	72 (64, 77)
VA	Male	60,068 (95.37)	50,723 (95.22)	51,941 (94.82)	47,080 (94.88)	46,879 (94.54)
VA	White	43,944 (70.29)	36,095 (68.51)	37,118 (68.56)	33,138 (67.64)	32,971 (67.46)
VA	Black	14,632 (23.40)	13,021 (24.71)	13,245 (24.46)	12,266 (25.04)	12,230 (25.02)
VA	Asian	282 (0.45)	300 (0.57)	295 (0.54)	271 (0.55)	286 (0.59)
VA	Hispanic	3,864 (6.15)	3,493 (6.59)	3,540 (6.50)	3,344 (6.77)	3,455 (7.01)
VA	Married	28,114 (44.64)	23,573 (44.25)	24,855 (45.37)	22,700 (45.75)	22,914 (46.21)
VA	Rural	18,323 (29.09)	14,712 (27.62)	15,290 (27.91)	13,249 (26.70)	13,280 (26.78)
VA	Charlson	4.80 (3.45)	4.99 (3.57)	4.99 (3.59)	5.12 (3.62)	5.19 (3.65)
VA	Hospital LOS	6 (3, 10)	6 (4, 11)	6 (4, 12)	6 (3, 11)	6 (3, 11)
VA	ICU LOS	2 (2, 4)	2 (2, 5)	3 (2, 5)	3 (2, 5)	3 (2, 5)
VA	ADI	59 (38, 80)	62 (39, 81)	61 (39, 81)	60 (38, 80)	60 (38, 80)
VA	CMI	1.53 (1.02, 2.15)	1.67 (1.09, 2.27)	1.68 (1.11, 2.21)	1.61 (1.10, 2.08)	1.60 (1.06, 2.08)
VA	Medicine CMI	1.24 (0.88, 1.71)	1.34 (0.94, 1.87)	1.34 (0.94, 1.85)	1.27 (0.93, 1.85)	1.27 (0.91, 1.77)
VA	Surgery CMI	2.74 (1.99, 4.15)	3.12 (2.05, 4.50)	3.06 (1.99, 4.61)	3.12 (2.03, 4.61)	3.00 (2.02, 4.60)
VA	HFD	89 (83, 90)	89 (80, 90)	89 (79, 90)	89 (81, 90)	89 (82, 90)
VA	Inhospital Mortality	3,681 (5.84)	5,165 (9.70)	5,568 (10.16)	4,013 (8.09)	3,521 (7.10)
VA	Medicine Inhospital Mortality	2,991 (4.75)	4,361 (8.19)	4,791 (8.75)	3,379 (6.81)	2,981 (6.02)

© 2025 Hahn Z et al. *JAMA Health Forum*.

Facility	Measure	2019	2020	2021	2022	2023
VA	Surgery Inhospital Mortality	661 (1.05)	761 (1.43)	724 (1.32)	596 (1.20)	509 (1.03)
VA	90-Day Mortality	10,519 (16.70)	11,235 (21.09)	11,740 (21.43)	9,710 (19.57)	9,020 (18.19)
CCN	Admissions	143,030	162,190	177,293	189,212	209,953
CCN	Medicine Admissions	96,443 (67.43)	112,270 (69.22)	122,817 (69.27)	130,700 (69.08)	144,925 (69.03)
CCN	Patients	114,205	129,415	142,615	149,734	163,185
CCN	Age	70 (62, 75)	71 (63, 76)	72 (63, 76)	73 (64, 77)	73 (65, 78)
CCN	Male	136,304 (95.30)	155,013 (95.57)	169,002 (95.32)	180,281 (95.28)	199,792 (95.16)
CCN	White	109,075 (77.48)	122,882 (77.04)	133,997 (77.01)	142,189 (76.66)	157,027 (76.56)
CCN	Black	21,014 (14.93)	23,988 (15.04)	25,951 (14.91)	27,433 (14.79)	30,677 (14.96)
CCN	Asian	661 (0.47)	800 (0.50)	923 (0.53)	963 (0.52)	1,163 (0.57)
CCN	Hispanic	6,415 (4.52)	7,286 (4.53)	8,298 (4.72)	8,782 (4.69)	9,854 (4.75)
CCN	Married	67,886 (47.46)	77,324 (47.67)	86,081 (48.55)	94,302 (49.84)	105,879 (50.43)
CCN	Rural	55,184 (38.58)	63,262 (39.00)	69,560 (39.23)	74,589 (39.42)	83,634 (39.83)
CCN	Charlson	4.03 (3.27)	4.15 (3.31)	4.10 (3.31)	4.25 (3.36)	4.33 (3.39)
CCN	Hospital LOS	4 (2, 8)	5 (2, 8)	5 (3, 9)	5 (3, 9)	5 (2, 8)
CCN	ICU LOS	3 (1, 5)	3 (2, 6)	3 (2, 6)	3 (2, 6)	3 (2, 6)
CCN	ADI	62 (41, 81)	63 (43, 82)	63 (43, 82)	63 (42, 81)	62 (42, 81)
CCN	CMI	1.69 (1.07, 2.45)	1.83 (1.15, 2.53)	1.85 (1.21, 2.65)	1.85 (1.23, 2.56)	1.77 (1.16, 2.56)
CCN	Medicine CMI	1.32 (0.92, 1.82)	1.35 (0.99, 1.87)	1.44 (1.02, 1.87)	1.38 (1.02, 1.87)	1.29 (1.00, 1.92)
CCN	Surgery CMI	3.49 (2.20, 5.06)	3.71 (2.40, 5.11)	3.79 (2.51, 5.18)	3.73 (2.52, 5.19)	3.78 (2.44, 5.08)
CCN	HFD	89 (83, 90)	89 (81, 90)	89 (78, 90)	89 (80, 90)	89 (81, 90)
CCN	Inhospital Mortality	7,664 (5.36)	11,924 (7.35)	15,286 (8.62)	13,854 (7.32)	12,996 (6.19)
CCN	Medicine Inhospital Mortality	5,624 (3.93)	9,362 (5.77)	12,098 (6.82)	10,699 (5.65)	9,877 (4.70)

© 2025 Hahn Z et al. *JAMA Health Forum*.

Facility	Measure	2019	2020	2021	2022	2023
CCN	Surgery Inhospital Mortality	2,023 (1.41)	2,542 (1.57)	3,174 (1.79)	3,137 (1.66)	3,100 (1.48)
CCN	90-Day Mortality	24,004 (16.78)	32,994 (20.34)	39,062 (22.03)	39,156 (20.69)	41,422 (19.73)

Abbreviations: ADI, Area Deprivation Index; CCI, Charlson Comorbidity Index; CMI, Case Mix Index (DRG-weight based); DRG, Diagnosis-Related Group; HFD90, Hospital-Free Days within 90 days; ICU, Intensive Care Unit; IQR, Interquartile Range; LOS, Length of Stay; VA, Veterans Affairs; VAMC, VA Medical Center; CCN, Community Care Network

© 2025 Hahn Z et al. *JAMA Health Forum*.

eTable 2. Admission Source by Facility Type

Admission Source	CCN (N, %)	VAMC (N, %)
Outpatient	721,631 (81.8)	249,541 (92.3)
Transfer	140,150 (15.9)	12,125 (4.5)
SNF	15,509 (1.8)	7,783 (2.9)
Other	309 (0.0)	560 (0.2)
Observation	135 (0.0)	143 (0.1)
Unknown	3,944 (0.4)	85 (0.0)
Total	881,678 (100)	270,237 (100)

Abbreviations: CCN, Community Care Network hospitals; VAMC VA Medical Center

eTable 3: Disposition at Hospital Discharge by Facility Type

Disposition Type	CCN (N, %)	VAMC (N, %)
Outpatient/Home	567,924 (64.4)	200,365 (74.1)
SNF	154,677 (17.5)	27,681 (10.2)
Death (in-hospital)	61,724 (7.0)	21,948 (8.1)
Transfer	45,910 (5.2)	11,445 (4.2)
Hospice	29,745 (3.4)	1,575 (0.6)
AMA	20,198 (2.3)	6,809 (2.5)
Other	1,393 (0.2)	398 (0.1)
Unknown	107 (0.0)	16 (0.0)
Total	881,678 (100)	270,237 (100)

Abbreviations: CCN, Community Care Network hospitals; VAMC VA Medical Center

© 2025 Hahn Z et al. *JAMA Health Forum*.

eTable 4. Medical ICU Admissions Costs, Length of Stay, Case Mix, and Mortality by Facility Type

	2019		2020		2021		2022		2023	
Measure	**VAMC**	**CCN**	**VAMC**	**CCN**	**VAMC**	**CCN**	**VAMC**	**CCN**	**VAMC**	**CCN**
Admissions (N)	43,513	96,443	38,520	112,270	39,596	122,817	36,152	130,700	35,842	144,925
Mean nominal Cost per Admission	NA	14,050	NA	15,393	NA	16,677	NA	16,375	NA	16,102
Mean adjusted Cost per Admission	NA	16,522	NA	17,861	NA	18,509	NA	16,961	NA	16,102
Median LOS (days, IQR)	6 (3, 9)	4 (2, 7)	6 (4, 11)	4 (2, 7)	6 (4, 11)	4 (2, 8)	6 (4, 11)	4 (2, 8)	6 (3, 10)	4 (2, 7)
Median ICU LOS (IQR)	2 (1, 4)	3 (1, 5)	2 (1, 4)	3 (2, 5)	2 (2, 4)	3 (2, 6)	2 (1, 4)	3 (2, 5)	2 (1, 4)	3 (2, 5)
Mean CMI (SD)	1.37 (0.79)	1.43 (0.82)	1.54 (1.02)	1.56 (1.00)	1.56 (1.02)	1.62 (1.11)	1.46 (0.84)	1.56 (1.05)	1.46 (0.94)	1.55 (1.14)
Median CMI (IQR)	1.2 (0.9, 1.7)	1.3 (0.9, 1.8)	1.3 (0.9, 1.9)	1.4 (1.0, 1.9)	1.3 (0.9, 1.9)	1.4 (1.0, 1.9)	1.3 (0.9, 1.9)	1.4 (1.0, 1.9)	1.3 (0.9, 1.8)	1.3 (1.0, 2.0)
Mean CCI (SD)I	5.0 (3.5)	4.2 (3.3)	5.1 (3.6)	4.3 (3.4)	5.1 (3.7)	4.2 (3.4)	5.2 (3.7)	4.4 (3.4)	5.3 (3.7)	4.5 (3.4)
In-hospital Mortality (%)	6.9	5.8	11.3	8.3	12.1	9.9	9.3	8.2	8.3	6.8
90d Mortality (%)	20.1	19.3	24.5	23.4	25.3	25.2	22.8	23.8	21.4	22.6

Abbreviations: VAMC, VA Medical Center; CCN, Community Care Network; LOS, Length of Stay; ICU, Intensive Care Unit; CMI, Case Mix Index; CCI, Charlson Comorbidity Index; NA: Not Applicable

© 2025 Hahn Z et al. *JAMA Health Forum*.

eTable 5: Surgical ICU Admissions, Nominal Costs, Length of Stay, Case Mix, and Mortality by Facility Type

	2019		2020		2021		2022		2023	
Measure	**VAMC**	**CCN**	**VAMC**	**CCN**	**VAMC**	**CCN**	**VAMC**	**CCN**	**VAMC**	**CCN**
Admissions (N)	18,786	45,526	14,117	48,914	14,496	53,480	12,900	57,369	13,149	63,782
Mean nominal cost per Admission	NA	41,091	NA	41,316	NA	42,955	NA	44,267	NA	44,881
Mean adjusted Cost per Admission	NA	48,319	NA	47,939	NA	47,672	NA	45,852	NA	44,881
Median LOS (days, IQR)	6 (3, 11)	6 (3, 11)	7 (3, 12)	6 (3, 11)	6 (3, 12)	6 (3, 12)	6 (3, 12)	6 (3, 12)	6 (3, 11)	6 (3, 11)
Median ICU LOS (IQR)	3 (2, 5)	3 (2, 7)	3 (2, 5)	3 (2, 7)	3 (2, 5)	4 (2, 8)	3 (2, 5)	4 (2, 8)	3 (2, 5)	3 (2, 7)
Mean CMI (SD)	3.39 (2.28)	4.26 (3.10)	3.66 (2.55)	4.40 (3.23)	3.66 (2.55)	4.49 (3.31)	3.63 (2.43)	4.43 (3.17)	3.56 (2.42)	4.39 (3.32)
Median CMI (IQR)	2.7 (2.0, 4.2)	3.5 (2.2, 5.1)	3.1 (2.0, 4.5)	3.7 (2.4, 5.1)	3.1 (2.0, 4.6)	3.8 (2.5, 5.2)	3.1 (2.0, 4.6)	3.7 (2.5, 5.2)	3.0 (2.0, 4.6)	3.8 (2.4, 5.1)
Mean CCI (SD)	4.4 (3.2)	3.7 (3.1)	4.8 (3.4)	3.9 (3.2)	4.8 (3.3)	3.9 (3.2)	4.8 (3.4)	4.0 (3.2)	4.8 (3.4)	4.0 (3.3)
In-hospital Mortality (%)	3.5	4.4	5.4	5.2	5.0	5.9	4.6	5.5	3.9	4.9
90d Mortality (%)	9.1	11.7	12.0	13.7	11.0	15.0	10.8	14.0	9.6	13.6

Abbreviations: VAMC, VA Medical Center; CCN, Community Care Network; LOS, Length of Stay; ICU, Intensive Care Unit; CMI, Case Mix Index; CCI, Charlson Comorbidity Index; NA: Not Applicable

© 2025 Hahn Z et al. *JAMA Health Forum*.

eTable 6. Interrupted Time Series (ITS) Regression Estimates for Monthly ICU Admissions, by Facility Type and DRG Group

Term	All DRGs Estimate (95% CI)	Medical DRGs Estimate (95% CI)	Surgical DRGs Estimate (95% CI)
**VA Level Pre-MISSION**	5,458.4 (4,512.0 to 6,404.8)	3,802.8 (3,078.7 to 4,526.9)	1,655.6 (1,235.7 to 2,075.5)
**VA Trend Pre-MISSION**	6.0 (−279.4 to 291.4)	5.4 (−212.9 to 223.7)	0.6 (−126.0 to 127.2)
**CCN Level Pre-MISSION**	10,914.9 (9,968.5 to 11,861.3)	7,550.6 (6,826.5 to 8,274.7)	3,364.3 (2,944.4 to 3,784.2)
**CCN Trend Pre-MISSION**	−52.7 (−338.1 to 232.7)	−76.0 (−294.3 to 142.3)	23.3 (−103.3 to 149.9)
**VA Level MISSION**	−404.3 (−1,555.6 to 747.1)	−321.4 (−1,202.2 to 559.4)	−82.9 (−593.6 to 427.9)
**VA Trend MISSION**	−12.0 (−128.5 to 104.5)	3.7 (−85.4 to 92.8)	−15.7 (−67.4 to 36.0)
**CCN Level MISSION**	−179.9 (−1,331.2 to 971.4)	−444.5 (−1,325.3 to 436.3)	264.6 (−246.2 to 775.3)
**CCN Trend MISSION**	466.1 (349.6 to 582.6)	360.9 (271.8 to 450.0)	105.3 (53.6 to 156.9)
**VA Level COVID**	−802.8 (−1,552.8 to −52.7)	−346.8 (−920.6 to 227.0)	−455.9 (−788.7 to −123.2)
**VA Trend COVID**	7.4 (−16.2 to 31.0)	4.3 (−13.8 to 22.3)	3.1 (−7.4 to 13.6)
**CCN Level COVID**	1,883.2 (1,133.2 to 2,633.3)	1,656.0 (1,082.2 to 2,229.8)	227.2 (−105.5 to 560.0)
**CCN Trend COVID**	117.7 (94.1 to 141.3)	84.6 (66.5 to 102.6)	33.1 (22.7 to 43.6)
**VA Level Post-COVID**	−159.9 (−715.3 to 395.6)	−114.2 (−539.1 to 310.7)	−45.7 (−292.1 to 200.7)
**VA Trend Post-COVID**	−1.0 (−36.0 to 34.0)	−1.8 (−28.5 to 25.0)	0.8 (−14.7 to 16.3)
**CCN Level Post-COVID**	2,621.0 (2,065.5 to 3,176.4)	1,731.8 (1,306.9 to 2,156.8)	889.1 (642.7 to 1,135.5)
**CCN Trend Post-COVID**	146.1 (111.1 to 181.1)	109.4 (82.6 to 136.1)	36.7 (21.2 to 52.2)

Abbreviations: VA, Veterans Affairs; CCN, Community Care Network; ICU, Intensive Care Unit; DRG, Diagnosis-Related Group; CI, Confidence Interval. Estimates derived from segmented interrupted time series regression models stratified by facility type and policy period. Level change reflects estimated intercept shift; trend change reflects estimated monthly slope difference.

© 2025 Hahn Z et al. *JAMA Health Forum*.

eTable 7. Interrupted Time Series (ITS) Regression Estimates for 90-Day Mortality, by Facility Type and DRG Group

Term	All DRGs<br>Estimate (95% CI)	Medical DRGs<br>Estimate (95% CI)	Surgical DRGs<br>Estimate (95% CI)
**VA Level Pre-MISSION**	17.65 (16.86 to 18.45)	21.25 (19.37 to 23.13)	9.41 (8.60 to 10.21)
**VA Trend Pre-MISSION**	−0.29 (−0.53 to −0.05)	−0.39 (−0.95 to 0.18)	−0.07 (−0.32 to 0.17)
**CCN Level Pre-MISSION**	17.24 (16.44 to 18.03)	19.59 (17.71 to 21.47)	11.97 (11.16 to 12.77)
**CCN Trend Pre-MISSION**	−0.14 (−0.38 to 0.10)	−0.16 (−0.73 to 0.40)	0.00 (−0.24 to 0.25)
**VA Level MISSION**	−2.38 (−3.35 to −1.42)	−2.50 (−4.79 to −0.21)	−1.85 (−2.83 to −0.87)
**VA Trend MISSION**	0.36 (0.26 to 0.46)	0.34 (0.11 to 0.57)	0.33 (0.23 to 0.43)
**CCN Level MISSION**	−1.84 (−2.81 to −0.87)	−1.87 (−4.16 to 0.42)	−1.10 (−2.08 to −0.12)
**CCN Trend MISSION**	0.37 (0.27 to 0.46)	0.41 (0.18 to 0.65)	0.18 (0.08 to 0.27)
**VA Level COVID**	5.87 (5.24 to 6.50)	5.51 (4.02 to 7.00)	5.03 (4.39 to 5.66)
**VA Trend COVID**	0.03 (0.01 to 0.05)	0.06 (0.02 to 0.11)	−0.08 (−0.10 to −0.06)
**CCN Level COVID**	4.55 (3.92 to 5.18)	5.11 (3.62 to 6.60)	2.49 (1.85 to 3.12)
**CCN Trend COVID**	0.13 (0.11 to 0.15)	0.14 (0.09 to 0.19)	0.09 (0.07 to 0.11)
**VA Level Post-COVID**	−2.70 (−3.16 to −2.23)	−3.09 (−4.19 to −1.98)	−1.71 (−2.19 to −1.24)
**VA Trend Post-COVID**	0.01 (−0.02 to 0.04)	0.04 (−0.03 to 0.11)	−0.07 (−0.10 to −0.04)
**CCN Level Post-COVID**	0.02 (−0.45 to 0.49)	0.22 (−0.89 to 1.32)	0.01 (−0.46 to 0.48)
**CCN Trend Post-COVID**	0.01 (−0.02 to 0.04)	−0.02 (−0.09 to 0.05)	0.04 (0.01 to 0.07)

Abbreviations: VA, Veterans Affairs; CCN, Community Care Network; ICU, Intensive Care Unit; DRG, Diagnosis-Related Group; CI, Confidence Interval.
Estimates derived from segmented interrupted time series regression models stratified by facility type and policy period. Level change reflects estimated intercept shift; trend change reflects estimated monthly slope difference.

© 2025 Hahn Z et al. *JAMA Health Forum*.

eTable 8. Interrupted Time Series (ITS) Regression Estimates for Monthly Case Mix Index, by Facility Type and DRG Group

Term	All DRGs Estimate (95% CI)	Medical DRGs Estimate (95% CI)	Surgical DRGs Estimate (95% CI)
**VA Level Pre-MISSION**	1.56 (1.46 to 1.66)	1.25 (1.07 to 1.42)	2.76 (2.51 to 3.00)
**VA Trend Pre-MISSION**	−0.02 (−0.05 to 0.01)	−0.01 (−0.06 to 0.05)	−0.01 (−0.08 to 0.07)
**CCN Level Pre-MISSION**	1.67 (1.57 to 1.77)	1.33 (1.15 to 1.50)	3.47 (3.23 to 3.72)
**CCN Trend Pre-MISSION**	0.01 (−0.02 to 0.04)	0.00 (−0.06 to 0.05)	0.01 (−0.07 to 0.08)
**VA Level MISSION**	−0.04 (−0.16 to 0.08)	−0.03 (−0.24 to 0.19)	−0.20 (−0.50 to 0.10)
**VA Trend MISSION**	0.00 (−0.01 to 0.01)	0.00 (−0.02 to 0.02)	0.04 (0.01 to 0.07)
**CCN Level MISSION**	−0.01 (−0.13 to 0.11)	−0.12 (−0.33 to 0.09)	−0.04 (−0.34 to 0.26)
**CCN Trend MISSION**	0.00 (−0.01 to 0.02)	0.02 (−0.01 to 0.04)	0.02 (−0.01 to 0.05)
**VA Level COVID**	0.16 (0.09 to 0.24)	0.11 (−0.02 to 0.25)	0.55 (0.36 to 0.75)
**VA Trend COVID**	0.00 (0.00 to 0.00)	0.00 (0.00 to 0.01)	0.00 (−0.01 to 0.00)
**CCN Level COVID**	0.16 (0.08 to 0.24)	0.16 (0.02 to 0.30)	0.24 (0.05 to 0.43)
**CCN Trend COVID**	0.00 (0.00 to 0.00)	0.01 (0.00 to 0.01)	0.01 (0.00 to 0.01)
**VA Level Post-COVID**	−0.08 (−0.13 to −0.02)	−0.08 (−0.18 to 0.02)	−0.02 (−0.16 to 0.13)
**VA Trend Post-COVID**	0.00 (0.00 to 0.00)	0.00 (−0.01 to 0.01)	0.00 (−0.01 to 0.01)
**CCN Level Post-COVID**	0.03 (−0.02 to 0.09)	−0.04 (−0.14 to 0.06)	0.02 (−0.12 to 0.17)
**CCN Trend Post-COVID**	−0.01 (−0.01 to 0.00)	0.00 (−0.01 to 0.01)	0.00 (0.00 to 0.01)

Abbreviations: VA, Veterans Affairs; CCN, Community Care Network; ICU, Intensive Care Unit; DRG, Diagnosis-Related Group; CI, Confidence Interval.
Estimates derived from segmented interrupted time series regression models stratified by facility type and policy period. Level change reflects estimated intercept shift; trend change reflects estimated monthly slope difference.

© 2025 Hahn Z et al. *JAMA Health Forum*.

eTable 9. Interrupted Time Series (ITS) Regression Estimates for Monthly Charlson Comorbidity Index, by Facility Type and DRG Group

Term	All DRGs<br>Estimate (95% CI)	Medical DRGs<br>Estimate (95% CI)	Surgical DRGs<br>Estimate (95% CI)
**VA Level Pre-MISSION**	4.63 (4.49 to 4.76)	4.81 (4.65 to 4.97)	4.19 (4.02 to 4.37)
**VA Trend Pre-MISSION**	0.03 (−0.01 to 0.07)	0.03 (−0.02 to 0.07)	0.03 (−0.02 to 0.08)
**CCN Level Pre-MISSION**	3.98 (3.85 to 4.11)	4.16 (4.00 to 4.32)	3.57 (3.39 to 3.74)
**CCN Trend Pre-MISSION**	0.01 (−0.03 to 0.05)	0.00 (−0.04 to 0.05)	0.03 (−0.02 to 0.08)
**VA Level MISSION**	0.15 (0.00 to 0.31)	0.11 (−0.08 to 0.31)	0.26 (0.05 to 0.47)
**VA Trend MISSION**	0.02 (0.01 to 0.04)	0.02 (0.01 to 0.04)	0.02 (0.00 to 0.04)
**CCN Level MISSION**	−0.01 (−0.17 to 0.15)	−0.03 (−0.22 to 0.17)	0.06 (−0.15 to 0.27)
**CCN Trend MISSION**	0.02 (0.01 to 0.04)	0.02 (0.00 to 0.04)	0.02 (0.00 to 0.04)
**VA Level COVID**	0.21 (0.11 to 0.32)	0.12 (−0.01 to 0.25)	0.38 (0.24 to 0.52)
**VA Trend COVID**	0.00 (0.00 to 0.00)	0.00 (0.00 to 0.01)	0.00 (−0.01 to 0.00)
**CCN Level COVID**	0.18 (0.08 to 0.29)	0.15 (0.02 to 0.28)	0.21 (0.07 to 0.35)
**CCN Trend COVID**	0.00 (0.00 to 0.00)	0.00 (−0.01 to 0.00)	0.00 (0.00 to 0.01)
**VA Level Post-COVID**	0.11 (0.03 to 0.18)	0.18 (0.08 to 0.27)	−0.08 (−0.18 to 0.02)
**VA Trend Post-COVID**	0.01 (0.00 to 0.01)	0.01 (0.00 to 0.01)	0.01 (0.00 to 0.01)
**CCN Level Post-COVID**	0.09 (0.01 to 0.16)	0.09 (0.00 to 0.18)	0.09 (−0.02 to 0.19)
**CCN Trend Post-COVID**	0.01 (0.00 to 0.01)	0.01 (0.00 to 0.01)	0.01 (0.00 to 0.01)

Abbreviations: VA, Veterans Affairs; CCN, Community Care Network; ICU, Intensive Care Unit; DRG, Diagnosis-Related Group; CI, Confidence Interval. Estimates derived from segmented interrupted time series regression models stratified by facility type and policy period. Level change reflects estimated intercept shift; trend change reflects estimated monthly slope difference.

© 2025 Hahn Z et al. *JAMA Health Forum*.

eTable 10. Nominal and Inflation-adjusted costs using the GDP Bureau of Economic Analysis Gross Domestic Product deflator

Year	GDP Price Index	Adjustment Factor	Nominal Cost Medicine	Nominal Cost Surgery	Inflation-Adjusted Cost Medicine	Inflation-Adjusted Cost Surgery	Total VA Spending (Nominal, B USD)	Total VA Spending (Inflation-Adjusted, B USD)
2019	103.98	1.18	890.8	1,405.1	1,047.6	1,652.3	2.30	2.70
2020	105.38	1.16	1,350.9	1,632.6	1,567.5	1,894.4	2.98	3.46
2021	110.17	1.11	1,585.1	1,782.3	1,759.2	1,978.0	3.37	3.74
2022	118.04	1.04	1,630.4	1,949.3	1,688.8	2,019.1	3.58	3.71
2023	122.27	1.00	1,803.3	2,239.7	1,803.3	2,239.7	4.04	4.04

Abbreviations: VA, Veterans Affairs; ICU, Intensive Care Unit; USD, US dollars; GDP, Gross Domestic Product. Costs reflect VA-purchased community ICU care. Inflation-adjusted values converted to constant 2023 USD using the GDP Price Index from the US Bureau of Economic Analysis.

© 2025 Hahn Z et al. *JAMA Health Forum*.

eTable 11. Absolute and Relative Missingness for Study Covariates by Year and Facility Type

Covariate	CCN-2019	VA-2019	CCN-2020	VA-2020	CCN-2021	VA-2021	CCN-2022	VA-2022	CCN-2023	VA-2023
**N**	143,030	62,982	162,190	53,268	177,293	54,781	189,212	49,622	209,953	49,584
**Age**	0 (0.00)	0 (0.00)	0 (0.00)	0 (0.00)	0 (0.00)	0 (0.00)	0 (0.00)	0 (0.00)	0 (0.00)	0 (0.00)
**Gender**	0 (0.00)	0 (0.00)	0 (0.00)	0 (0.00)	0 (0.00)	0 (0.00)	0 (0.00)	0 (0.00)	0 (0.00)	0 (0.00)
**Race**	2,260 (1.58)	461 (0.73)	2,682 (1.65)	582 (1.09)	3,289 (1.86)	638 (1.16)	3,725 (1.97)	629 (1.27)	4,860 (2.31)	709 (1.43)
**Ethnicity**	993 (0.69)	183 (0.29)	1,241 (0.77)	277 (0.52)	1,603 (0.90)	315 (0.58)	1,847 (0.98)	258 (0.52)	2,386 (1.14)	297 (0.60)
**Rurality**	1,049 (0.73)	460 (0.73)	933 (0.58)	571 (1.07)	957 (0.54)	558 (1.02)	1,042 (0.55)	434 (0.87)	1,311 (0.62)	447 (0.90)
**CMI**	252 (0.18)	553 (0.88)	294 (0.18)	870 (1.63)	49 (0.03)	564 (1.03)	49 (0.03)	726 (1.46)	2,462 (1.17)	567 (1.14)
**CCI**	0 (0.00)	0 (0.00)	0 (0.00)	0 (0.00)	0 (0.00)	0 (0.00)	0 (0.00)	0 (0.00)	0 (0.00)	0 (0.00)
**Hospital LOS**	0 (0.00)	0 (0.00)	0 (0.00)	0 (0.00)	0 (0.00)	0 (0.00)	0 (0.00)	0 (0.00)	0 (0.00)	0 (0.00)
**ICU LOS**	0 (0.00)	0 (0.00)	0 (0.00)	0 (0.00)	0 (0.00)	0 (0.00)	0 (0.00)	0 (0.00)	0 (0.00)	0 (0.00)

Abbreviations: CCN, Community Care Network; VA, Veterans Affairs; CMI, Case Mix Index; CCI, Charlson Comorbidity Index; LOS, Length of Stay; ICU, Intensive Care Unit.

© 2025 Hahn Z et al. *JAMA Health Forum*.

eTable 12. VAMC ICU Admission rates by DRG Group and Year

Year	DRG Group	Median VAMC ICU Admission Rate (IQR), %
2019	All	16.08 (12.02 to 19.17)
2020	All	15.77 (12.49 to 19.33)
2021	All	15.26 (12.30 to 18.15)
2022	All	14.87 (11.72 to 18.02)
2023	All	14.97 (11.52 to 18.43)
2019	Medicine	13.42 (10.47 to 17.90)
2020	Medicine	14.30 (11.15 to 17.76)
2021	Medicine	13.31 (10.71 to 16.87)
2022	Medicine	13.23 (10.41 to 16.39)
2023	Medicine	12.78 (9.60 to 17.57)
2019	Surgery	24.12 (16.33 to 29.87)
2020	Surgery	24.54 (17.64 to 30.16)
2021	Surgery	22.37 (15.41 to 29.22)
2022	Surgery	22.21 (14.53 to 28.42)
2023	Surgery	21.46 (14.09 to 30.05)

Abbreviations: VA, Veterans Affairs; ICU, Intensive Care Unit; IQR, Interquartile Range.
ICU admission rates are expressed as percentages, defined as the proportion of hospital admissions resulting in ICU care. Median rates and interquartile ranges were calculated at the facility level for each year and DRG group.

© 2025 Hahn Z et al. *JAMA Health Forum*.

eTable 13. VA-Purchased ICU Bed-Days as a Proportion of National Non-Medicare/Medicaid ICU Use and Trends in ICU Occupancy, 2019–2023

Year	Community ICU Bed-Days Available[a]	VAMC ICU Bed-Days Available[b]	Non-Medicare/Medicaid Bed-Days[c] N (%)	VA purchased Bed-Days[d] N (%)	VAMC delivered Bed-Days[e], N (%)	VAMC Licensed ICU Occupancy[f], Median, (IQR)	Licensed ICU Occupancy[g] Median, (IQR)	Staffed ICU Occupancy[h] Median, (IQR)
2019	25,771,946	702,990	5,686,862 (22.1)	662,091 (11.6)	238,802 (34.0)	51.5 (51.1, 52.1)	55.8 (40.0, 71.3)	Not available
2020	26,609,233	812,125	6,099,109 (22.9)	797,322 (13.1)	230,381 (28.4)	46.1 (42.8, 48.4)	59.2 (42.9, 74.1)	80.0 (66.3, 93.8)
2021	27,160,543	841,325	6,580,766 (24.2)	941,038 (14.3)	240,640 (28.6)	45.0 (44.4, 46.5)	64.0 (47.8, 78.7)	83.6 (69.5, 96.9)
2022	27,241,143	791,685	6,109,258 (22.4)	970,672 (15.9)	207,837 (26.3)	49.3 (47.0, 51.2)	59.2 (42.7, 74.8)	82.9 (67.1, 96.7)
2023	27,088,819	727,810	5,913,393 (21.8)	1,014,921 (17.2)	204,355 (28.1)	48.6 (48.2, 49.0)	56.2 (38.8, 72.9)	81.1 (65.0, 94.9)

Abbreviations: CCN, Community Care Network; ICU, intensive care unit; IQR, interquartile range; VAMC, Veterans Affairs Medical Center.

[a] Total annual ICU bed-days available in community hospitals, derived from RAND Hospital Data based on reported licensed ICU bed capacity across U.S. hospitals.

[b] Total annual ICU bed-days available at VAMCs, calculated as the number of operating ICU beds × 365 days/year.

[c] Annual ICU bed-days in community hospitals attributed to patients not covered by Medicare or Medicaid, from RAND data. The percentage reflects the share of total community ICU bed-days.

[d] VA-purchased ICU bed-days in community hospitals through the Community Care Network (CCN); percentage reflects share of non-Medicare/Medicaid ICU bed-days.

[e] VAMC-delivered ICU bed-days based on VA CDW data. Percentage reflects share of available VAMC ICU bed-days that were occupied annually.

[f] VAMC ICU occupancy calculated as the median monthly percentage of licensed beds occupied within VA medical centers; interquartile range (IQR) shown in parentheses.

[g] National ICU occupancy based on licensed bed capacity from RAND data, restricted to community hospitals caring for Veterans under CCN.

[h] National ICU occupancy based on staffed beds from CDC/HHS Protect data, reflecting functional ICU bed capacity in community hospitals caring for Veterans under CCN.

© 2025 Hahn Z et al. *JAMA Health Forum*.

eFigure 1. Cohort derivation for VA-purchased or delivered ICU admissions between 2016 and 2023.

Flow diagram depicting the derivation of the final analysis cohort from an initial pool of 1,182,937 ICU admissions. Records were excluded based on the following criteria: minor status (N = 9), newborn status (N = 11), missing Diagnosis-Related Group (DRG) codes (N = 15,238), zero ICU days (N = 14,665), or negative days to death (N = 1,099). The resulting final cohort (N = 1,151,915) was stratified by site of care: Veterans Affairs Medical Centers (VAMC; N = 270,237) and Community Care Network (CCN; N = 881,678).

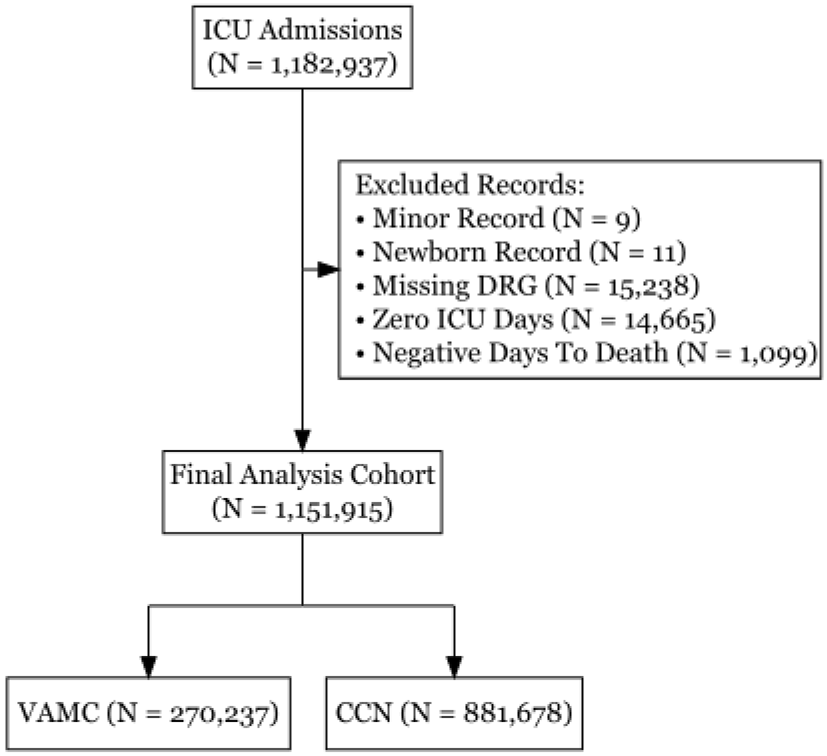

© 2025 Hahn Z et al. *JAMA Health Forum*.

eFigure 2. Interrupted time-series trends in veteran medical intensive care utilization and outcomes, 2019-2023

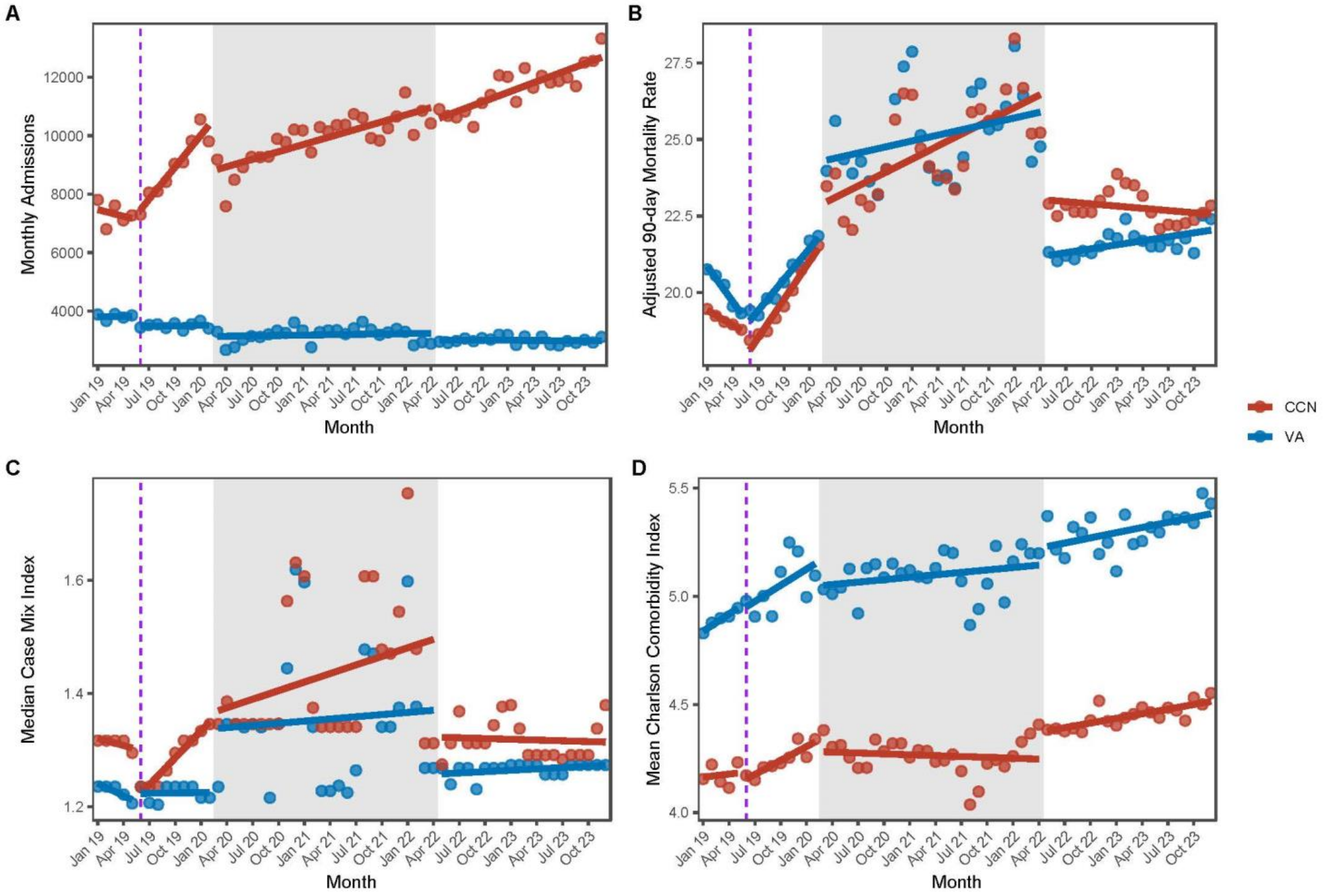

A, ICU admissions. B, 90-day mortality. C, Case-Mix Index. D, Charlson Comorbidity Index. Dashed vertical line = VA MISSION Act implementation (June 2019); gray band = COVID-19 emergency (March 2020 – April 2022).

Abbreviations: CCN, Community Care Network; VA, Veterans Affairs. Trend lines are model-based; dots represent monthly observations.

© 2025 Hahn Z et al. *JAMA Health Forum*.

eFigure 3. Interrupted time-series trends in veteran surgical intensive care utilization and outcomes, 2019-2023

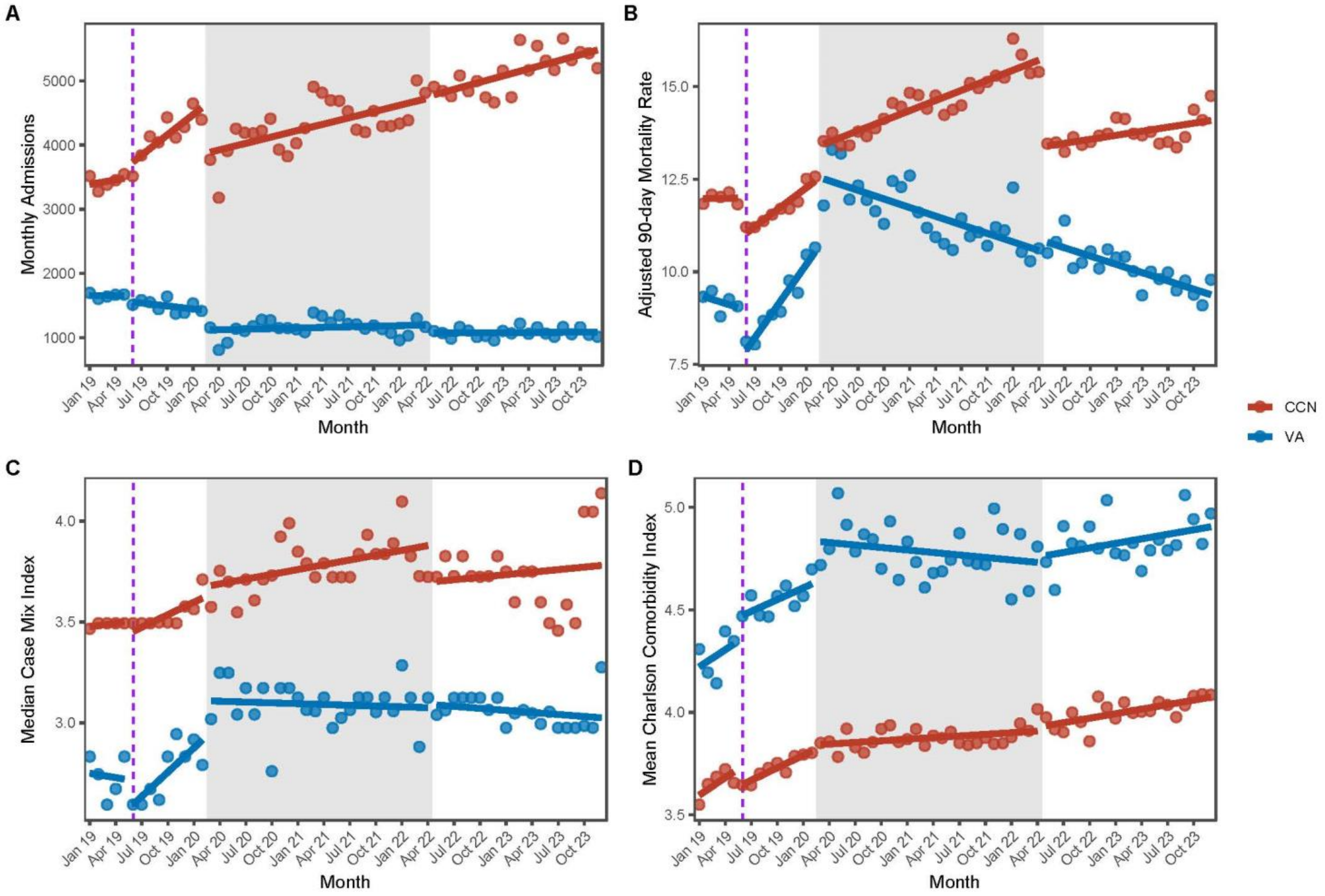

A, ICU admissions. B, 90-day mortality. C, Case-Mix Index. D, Charlson Comorbidity Index. Dashed vertical line = VA MISSION Act implementation (June 2019); gray band = COVID-19 emergency (March 2020 – April 2022).

Abbreviations: CCN, Community Care Network; VA, Veterans Affairs. Trend lines are model-based; dots represent monthly observations.

© 2025 Hahn Z et al. *JAMA Health Forum*.
