# Supplementary material for: Intensive Care Unit Admissions Purchased or Delivered by Veterans in the VA Health Care System
Source: JAMA Health Forum. 2025 Dec 12;6(12):e255605. doi: 10.1001/jamahealthforum.2025.5605 (PMC12701510; doi:10.1001/jamahealthforum.2025.5605)
Supplement: Supplement 2. — Data Sharing Statement [file jamahealthforum-e255605-s002.pdf]

Data Sharing Statement

Hahn. Intensive Care Unit Admissions Purchased or Delivered by Veterans in the VA Health Care System. *JAMA Health Forum*. Published December 12, 2025.
doi:10.1001/jamahealthforum.2025.5605

Data
Data available: No

Additional Information
Explanation for why data not available: The data that supports this study's findings are available from the VA. VA data with an approved VA study protocol is freely available to researchers behind the VA firewall. More information is available at https://www.virec.research.va.gov or the VA Information Resource Center (VIReC) at VIReC@va.gov.
